# Monoclonal Antibodies as Neurological Therapeutics

**DOI:** 10.3390/ph14020092

**Published:** 2021-01-26

**Authors:** Panagiotis Gklinos, Miranta Papadopoulou, Vid Stanulovic, Dimos D. Mitsikostas, Dimitrios Papadopoulos

**Affiliations:** 1 Department of Neurology, KAT General Hospital of Attica, 14561 Athens, Greece; gklinosp@gmail.com; 2 Center for Clinical, Experimental Surgery & Translational Research, Biomedical Research Foundation of the Academy of Athens (BRFAA), 11527 Athens, Greece; mirpapadopoulou11@gmail.com; 3 Global Pharmacovigilance, R&D Sanofi, 91385 Chilly-Mazarin, France; vid.stanulovic@sanofi.com; 4 1st Neurology Department, Aeginition Hospital, National and Kapodistrian University of Athens, 11521 Athens, Greece; dmitsikostas@med.uoa.gr; 5 Laboratory of Molecular Genetics, Hellenic Pasteur Institute, 129 Vasilissis Sophias Avenue, 11521 Athens, Greece; 6 Salpetriere Neuropsychiatric Clinic, 149 Papandreou Street, Metamorphosi, 14452 Athens, Greece

**Keywords:** monoclonal antibodies, multiple sclerosis, migraine, neuromyelitis optica spectrum disorder, myasthenia gravis, Alzheimer’s disease, inflammatory myopathies, immune-mediated peripheral neuropathies, Parkinson’s disease, neurooncology, Duchene’s muscular dystrophy

## Abstract

Over the last 30 years the role of monoclonal antibodies in therapeutics has increased enormously, revolutionizing treatment in most medical specialties, including neurology. Monoclonal antibodies are key therapeutic agents for several neurological conditions with diverse pathophysiological mechanisms, including multiple sclerosis, migraines and neuromuscular disease. In addition, a great number of monoclonal antibodies against several targets are being investigated for many more neurological diseases, which reflects our advances in understanding the pathogenesis of these diseases. Untangling the molecular mechanisms of disease allows monoclonal antibodies to block disease pathways accurately and efficiently with exceptional target specificity, minimizing non-specific effects. On the other hand, accumulating experience shows that monoclonal antibodies may carry class-specific and target-associated risks. This article provides an overview of different types of monoclonal antibodies and their characteristics and reviews monoclonal antibodies currently in use or under development for neurological disease.

## 1. Introduction

The production of monoclonal antibodies (mAbs) was first described in 1975 when Köhler and Milstein developed methods for their isolation from hybridoma cells [1]. The ability to generate mAbs revolutionized antibody research and paved the way for tremendous clinical advances. For their discovery, Milstein and Köhler shared the 1984 Nobel Prize for Medicine or Physiology together with Niels K. Jerne for “theories concerning the specificity in development and control of the immune system and discovery of the principle for production of monoclonal antibodies”. According to the classical hybridoma method, mice were immunized with a mixture of antigens, their antibody-producing splenic B cells were fused with immortalized neoplastic B cells (myeloma cells) bearing a selection marker and the fused cells (hybridoma cells) were cultured in a selective medium. When visible colonies grew, their supernatants were screened for antibody production. For the first time, unlimited amounts of monoclonal antibodies specific for a single determinant could thus be produced in vitro. Köhler and Milstein did not patent their method, which facilitated the use of hybridoma technology by academics and the pharmaceutical industry for the generation of future potential therapies. At first, myeloma cells which retained the capacity to secrete their own immunoglobulin products were used. Later, such fusion was replaced by myeloma variants that express only one endogenous chain so that the fused cells secreted primarily or exclusively the antibody of the desired specificity. Besides their huge impact on research and diagnostic applications including epitope-specific immunoblotting, immunofluorescence, and immunohistochemistry, mAbs played an important role in therapeutics, contributing to the treatment of cancer, autoimmune and infectious diseases.

The first mAb approved by FDA for human use was a murine anti-CD3 monoclonal antibody, muromonab (OKT3), used for the treatment of organ transplant rejection [2]. However, murine mAb-associated allergic reactions (immune reaction against proteins from different species) led to the development of chimeric antibodies in 1984 [3]. Chimeric mouse-human antibodies were produced by grafting the entire antigen-specific domain of a mouse antibody onto the constant domains of a human antibody using recombinant DNA techniques [3]. Rituximab, a mouse-human chimeric mAb against the B-cell lineage marker CD20 was the first to be approved in 1997 by FDA for the treatment of relapsed or refractory, CD20-positive, B-cell, low-grade or follicular non-Hodgkin’s lymphoma [4]. Humanization of murine mAbs was achieved in the second half of the 1980s using CDR grafting methodology [5]. Later, the development of fully human monoclonal antibodies, in which both the variable region (Fab) and the constant region (Fc) are 100% human, was made possible through the advent of in vitro phage display technology and the generation of different mouse strains expressing human variable domains. Advanced antibody engineering technologies, such as phage display, affinity maturation, single B cell antibody technology and human antibody mouse are described in detail by Lu et al. [6]. The development of biosimilar mAbs has in many cases decreased the cost of treatment.

Antibodies of all types (murine, chimeric, humanized and human) have been approved by Food and Drug Administration (FDA), European Medicines Agency (EMA) and other national agencies for the treatment of several diseases. Since the approval of OKT3, the use of mAbs has progressively come to dominate therapeutics in all fields of medicine, including neurology. Many of the mAbs used in neurology today have been repurposed from their original indications for hematological neoplasias (e.g., alemtuzumab, ofatumumab and rituximab) or rheumatological disease (e.g., tocilizumab) [4,7,8,9]. Other mAbs have been developed originally for neurological disease (e.g., ocrelizumab for multiple sclerosis or mAbs for migraine prophylaxis). Sixteen marketed mAbs are used in neurology primarily for neuroimmunological conditions and migraine (Table 1). Nevertheless, many more mAbs are in development for neuroimmunological and neurodegenerative conditions (Table 2). In this review we discuss some key features of mAbs and provide an overview of the mAbs used in neurological diseases.

## 2. Nomenclature

The nomenclature of the mAb reflects features such as proposed target, original host, modifications, and conjugation to other molecules. The International Nonproprietary Name (INN) guidelines published by the WHO in 2014 and 2017 describe the classification for mAb names [140,141]. The mAb names consist of a prefix, two substems (reduced to one substem in the 2017 document), and a suffix. The prefix is referred to as “random”; it is intended to provide a unique drug name. The substems designate the target (e.g., “ci” for cardiovascular, “so” for bone, “tu” for tumor) and the source (host) in which the antibody was originally produced (e.g.,”-o-” for murine “-xi-” for chimeric, “-zu-” for humanized, ”-nu-” for fully human). The second substem (which specifies the source of the antibody and whether it is humanized or chimeric) was eliminated in 2017 [8]. This change only applies to mAb created after 2017. The suffix for all mAbs is “mab.” Biosimilar mAbs are named as the reference drug followed by a four-letter suffix consisting of four unique and meaningless lowercase letters and separated from the reference name by a hyphen [142].

## 3. Basic Categories of Monoclonal Antibodies

### 3.1. Murine Antibodies

Murine antibodies are produced entirely from mouse protein and are the earliest mAbs developed. Due to the source of their production, they were recognized as allogeneic proteins, thus leading to polyclonal human anti-mouse antibody (HAMA) reactions, usually 2–3 weeks after their initial infusion [143]. HAMAs frequently had neutralizing action leading to rapid murine antibody inactivation or affected their pharmacokinetics promoting accelerated plasma elimination [144,145]. No murine mAb is currently in use in neurology.

### 3.2. Chimeric Antibodies

The serious limitations murine antibodies impose upon their clinical use, necessitated the development of new products with human components. Initially, the Fc portion of the antibody molecule, which dictates the functions of the antibody, was chemically exchanged with a human constant portion [146], giving rise to chimeric monoclonal antibodies. Chimeric mAbs contain 34% mouse protein in the variable region of the antibody, thus leading to a lower incidence of HAMA reactions compared to murine mAbs. Moreover, chimeric mAbs have a wide range of antigen specificities, increased cellular toxicity, and a beneficial pharmacokinetic and pharmacodynamic profile (longer half-life and increased affinity for the antigen) [147]. Rituximab and infliximab are the only chimeric mAbs currently in use in neurology (Table 1).

### 3.3. Humanized Antibodies

Advances in methods of molecular biology led to the development of humanized mAbs, which are 90% human, and only 10% mouse protein. Humanized mAbs are even less immunogenic compared to chimeric mAbs. Molecular techniques were used to further eliminate regions in the murine immunoglobulin chains that are not involved in the binding of antigen and to replace them with the corresponding human sequences. Complementarity-determining regions (CDRs) within the variable regions of both the heavy and light chains are of great importance in the binding specificity of the antibody. DNA fragments that correspond to the CDRs were grafted into the framework of human immunoglobulin genes using molecular methods [5]. Furthermore, replacement of some amino acid residues in the constant regions with the corresponding amino acids of the mouse “parental” monoclonal antibody proved advantageous [148]. Humanized antibodies retain the specificity and binding affinity of the “parental” murine mAbs, while being less immunogenic and acquiring biological functions of choice [149]. The great majority of mAbs in use or in development for neurological indications are humanized mAbs (Table 1 and Table 2).

### 3.4. Fully Human Monoclonal Antibodies

**Figure 1:**
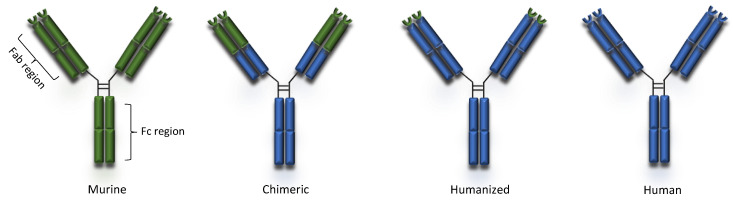
Types of monoclonal antibodies.

Peripheral blood lymphocytes or single cells derived from naïve and immunized donors were used to isolate immunoglobulin genes and to prepare libraries of plasmids with the cDNA’s of heavy and light chains. The combinatorial libraries were used to transfect bacteria which, in turn, were seeded on appropriate drug-supplemented agar medium Colonies producing active antibodies were then detected and isolated [150]. Phage display and transgenic mice technologies made production of 100% human mAbs possible [6]. Complete removal of murine components led to the production of mAbs that were mostly less immunogenic and, in many cases, improved their pharmacokinetic profiles slowing their clearance from plasma [147]. Erenumab and ofatumumab are fully human mAbs currently indicated for migraine prophylaxis and multiple sclerosis, respectively (Table 1). The human and murine components of murine, chimeric, humanized and human mAbs is schematically presented in Figure 1.

## 4. Mechanism of Action

Mabs may act through several direct and indirect mechanisms and some MAbs confer multiple mechanisms of action on a target [151].

### 4.1. Direct Mechanisms

Direct actions include antagonism of a soluble ligand or receptor, blockade of cell–cell interaction, agonism on a surface receptor activating certain signaling pathways within the target cell or inducing cell death [152,153]. The simplest form of antibody activity occurs when the antibody binds a soluble ligand, a cell-bound ligand, or a cell receptor, and blocks the binding of the ligand to the receptor, thereby disrupting the downstream signaling mediated by that receptor–ligand interaction. Examples of this activity is the binding of fremanezumab, galcanezumab and eptinezumab to the calcitonin gene-related peptide (CGRP) preventing it from signaling through the CGRP and Amylin-1 receptors [154,155].

Another approach is binding to a cell receptor in a non-agonistic manner to block ligand binding and activation of downstream signaling pathways as in the case of erenumab, which is an anti-CGRP receptor mAb [155]. Finally, cell–cell interactions between a cell-bound ligand and a cell-bound receptor on another cell can be blocked by mAbs, as in the case of natalizumab blocking lymphocytic transendothelial migration by binding to lymphocytic VLA-4 (CD49d) and preventing its binding to endothelial vascular cell adhesion molecule (VCAM) [55].

Agonistic mAbs mimic the activity of the normal ligand [151,156]. The agonist activity can occur when the antibody binds the receptor in a manner that mimics the binding of the natural ligand, resulting in antibody-mediated downstream signaling [156]. Alternatively, mAbs exerting agonist activity on receptors such as the tumor necrosis factor related apoptosis-inducing ligand (TRAIL) receptors initiate programmed cell death [157].

### 4.2. Indirect or Immune-Mediated Actions

**Figure 2:**
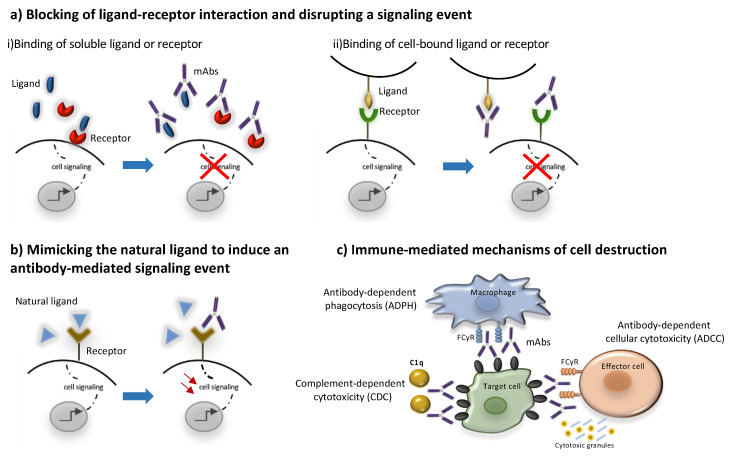
Mechanisms of action of monoclonal antibodies. MAbs may act through direct (**a**,**b**) or indirect mechanisms (**c**). The direct mechanisms include: (**a**) blocking ligand-receptor interactions through binding to (**i**) a soluble ligand or receptor or (**ii**) to a cell-bound ligand or receptor leading to inhibition of downstream signaling events, (**b**) agonism through binding to a receptor by mimicking its natural ligand leading to the activation of signaling pathways. Indirect mechanisms are immune -mediated as they involve the activation of certain types of immune cells and molecules to kill target cells (**c**)**.** Most mAbs bear a human IgG1 Fc region that can activate effector cells, such as natural killer (NK) cells to induce antibody-dependent immune cell cytotoxicity (ADCC), or macrophage inducing antibody-dependent phagocytosis (ADPH), through the interaction with their FCγ receptors. Moreover, the Fc region of mAbs can activate the complement leading to complement-dependent cytotoxicity (CDC).

Conserved differences in the constant regions (Fc) of IgG antibodies distinguish them into four subclasses: IgG1, IgG2, IgG3, and IgG4 [158,159]. These Fc regions are involved in binding to Fc receptors (FcγR), complement factor component 1q (C1q) and the neonatal receptor (FcRn) and as a result they determine the ability of different IgG subclasses to mediate effector functions such as phagocytosis, antibody-dependent cell-mediated cytotoxicity, complement activation and determine their half-life and capacity for transplacental transport and transport through mucosal surfaces [159] Most unconjugated antibodies bear a human IgG1 Fc, an isotype that efficiently activates the immune system, with the scope of harnessing different immune cells and molecules towards target cell killing. Thus, IgG1 mAbs may activate natural killer (NK) cells through CD16A, induce antibody-dependent cytotoxicity (ADCC), bind to macrophage CD16A, CD32A and CD64 to promote antibody-dependent phagocytosis (ADPh) and activate the complement leading to complement-dependent cytotoxicity (CDC) [158]. More specifically, to trigger ADCC, the Fc binding domain of an antibody binds to a specific antigen expressed on the surface of a target cell. The antibody is then able to recruit NK cells to lyse the target cell [150]. CDC is triggered when the C1 complement factor binds an IgG_1_ or IgG_3_ antibody–antigen complex, resulting in the activation of the complement cascade culminating in the formation of the C5b-9 membrane attack complex (MAC) forming a water pore in the target cell leading to its lysis [160]. Most of the marketed mAbs such as alemtuzumab and rituximab belong to the IgG1 subclass and are shown to trigger ADCC and CDC [73,161]. The immune mediated mode of action of mAbs is schematically presented in Figure 2. On the other hand, IgG2 and IgG4 subclasses exhibit a lower affinity to the Fcγ receptor and are commonly preferred for blocking antigen function. More specifically, the IgG2 subclass is commonly selected to neutralize soluble antigens without inducing host effector mechanisms as in the case of erenumab and fremanezumab [154,155]. Similarly, IgG4 such as natalizumab and galcanezumab represent an important subclass of mAbs commonly selected when the recruitment of the host effector mechanisms is not desirable [55,155,159,162].

### 4.3. Conjugated mAbs

Conjugated mAbs are combined with a drug or a radioactive substance. These mAbs are currently used in oncology to deliver these substances directly to cancer cells [163]. They are specifically designed to induce either a block in proliferation or direct cell death (usually apoptosis) and can deliver higher concentrations of cytotoxic agents directly to the target cells without affecting normal cells, thus reducing the potential of adverse reactions [158]. Ibritumomab tiuxetan is an example of a radiolabeled mAb against CD20, (a B cell surface protein), which is conjugated with radioactive Yttrium-90 and used in radioimmunotherapy and Ado-trastuzumab emtansine (also called TDM-1), is an antibody that targets the HER2 protein conjugated to a chemotherapeutic drug called DM1 [164,165]. Although conjugated mAbs have neither clinical nor experimental application in neurology, they could be used in the future to destroy targets or traffic medications to specific cell types.

### 4.4. Bispecific Monoclonal Antibodies

Bispecific mAbs are especially designed to recognize and bind to two epitopes simultaneously. Their unique structure confers them an unlimited potential of novel functions. Combining the two distinct binding sites in a single molecule yields a compound function that is restricted both in space and time, which cannot be achieved by the administration of a mixture of two separate mAbs with the same specificity. Bispecific Abs can direct effectors cells to target cells, promote receptor internalization, deliver ligands to specific cell populations, simultaneously block two pathways or promote shuttling across biological barriers [166]. The latter is particularly relevant to neurology where the blood-barrier barrier (BBB) is an obstacle for access of mAbs to the CNS. One specificity of a bispecific Abs can be used to shuttle it through the BBB (e.g., binding to the transferrin receptor) and the second specificity can bind to protein targets to block or promote a process or destroy brain tumor cells [167].

Two bispecific Abs are currently marketed and many other are in development. As an example blinatumomab, which is indicated for Philadelphia chromosome-negative relapsed or refractory acute lymphoblastic leukemia binds simultaneously to the CD3 protein of T cells and to the CD19 protein of target neoplastic B cells. By binding to both proteins, it brings T effector cells in close proximity to target neoplastic cells promoting their immune-mediated lysis [168]. Emicizumab is another bispecific Ab approved in EU and US for Hemophilia A as it binds simultaneously coagulation factors IXa and X [169]. Many more other bispecific Abs are in clinical development for several uses [168]. No bispecific Abs are currently in use in neurological therapeutics. However, preclinical evidence hold promise for their use in neurology in the future. Delivery of the construct of a bispecific Ab with an LDLR-binding domain of apoB to facilitate its transfer across the BBB and promoting alpha secretase activity over beta-secretase activity thus favoring the neuroprotective APP cleavage by alpha-secretase using an adenoviral vector has shown beneficial effects in a mouse model of AD [170]. In addition, targeting simultaneously the angiogenic factor angiopoietin-2 (Ang-2) and translocator protein (TSPO), both of which are overexpressed in bevacizumab-treated glioblastomas, with a bispecific Ab in bevacizumab-treated rats resulted in prolonged survival [171]. Furthermore, another bispecific Ab targeting Ang-2 and vascular endothelial growth factor (VEGF) was also found to prolong survival in a mouse model with glioblastoma xenografts, suggesting that bispecific Abs targeting appropriate epitopes may be beneficial in neurooncology [172].

## 5. Doses, Routes of Administration and Pharmacokinetics

Regarding dosing, some mAbs are given in a fixed dose whilst others are given according to patient’s bodyweight. MAbs require parenteral administration for adequate bioavailability. In most cases mAbs are administered either intravenously (e.g., natalizumab) or subcutaneously (e.g., eremumab). Some can be administered by either route (e.g., rituximab), whilst intramuscular administration has also been reported (e.g., palivizumab). Intravenous administration is chosen for greater and faster bioavailability and lower risk of immunogenicity whilst subcutaneous use is chosen to avoid intravenous access and facilitate self-administration [147,173]. Subcutaneously administered antibodies are taken up by lymphatics and their plasma concentration increase slowly over several days. Circulating mAbs leave the vasculature by hydrostatic and osmotic pressure gradients. Their affinity for the epitope of their specificity determines their retention in target tissues [173].

The half-lives of mAbs vary from hours to several weeks [174]. MAb half-life is largely determined by the binding of the constant fragment (Fc) of humanized and human Abs of immunoglobulin G (IgG) class to the neonatal receptor FcRn, expressed on many adult cell types [147]. More specifically, IgG antibodies are thought to be taken up by catabolic cells by fluid-phase endocytosis. Although, under neutral pH, FcRn has a low affinity for IgG, the endosome content is then acidified, thus increasing the affinity of the FcRn for IgG. The FcRn-IgG complex is then re-shuttled to the cell surface where the IgG is released under neutral pH [175]. Proteins and antibodies in the endosome that are not bound to the FcRn undergo proteolysis. This is a salvage pathway recycling and protecting IgGs from degradation therefore increasing their half-life without affecting their function. The half-life of IgG1, IgG2 and IgG4 is in the range of 18 to 21 days whereas the half-life of other proteins with comparable molecular weight is significantly shorter. The half-life of IgG3 mAbs, which have a lower affinity for the FcRn is approximately 7 days. Mabs which are Fc-deficient typically have an even shorter plasma half-life (e.g., 1.25 ± 0.63 h for blinatumomab in vivo), as they lack protection from degradation by the neonatal Fc receptor (FcRn) and in some cases also have a lower molecular weight than IgG, further increasing elimination through the kidneys [147,174]. It is conceivable that mAb internalization and FcRn-regulated release may affect the efficacy of a mAb if the dose of administration does not ensure that its free circulating fraction suffices to exert its action. Accordingly, blockade of the FcRn is therapeutically exploited to reduce the activity of pathogenic auto-antibodies (see rozanolixizumab, nipocalimab, batoclimab and efgartigimod in Section 6.5). A method to increase mAb half-life is to covalently attach a polyethylene glycol (PEG) chain to the mAb molecule (pegylation) as in the case of certolizumab pegol used for rheumatoid arthritis and Crohn’s disease [176].

The duration of biologic activity may differ substantially from their half-life because the former is primarily determined by the duration of the biological effects (e.g., the time required for a depleted cell population to recover). Consequently, the frequency of the administration depends on the mAb, its individual properties and the therapeutic strategy. Generally, mAbs are administered at fixed intervals, though in some cases dosing frequency may be determined by the duration of the effect as in the case of B cell depletion with rituximab treatment in multiple sclerosis (MS) and neuromyelitis optica spectrum disorders (NMOSD), where the peripheral blood CD19+ population may be used as a surrogate marker of B cells repopulation [177].

**Figure 3:**
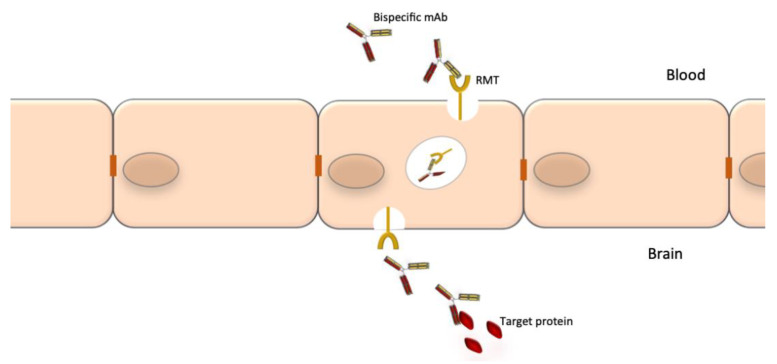
Crossing of the blood-brain barrier by specifically engineered bispecific mAbs. The blood-brain barrier (BBB) is an obstacle for the delivery of mAbs to the CNS. Bispecific mAbs may be used to cross the BBB as through their one specificity they can be shuttled via receptor-mediated transport (RMT), while the other can be used to bind the target proteins.

A notable drawback of using mAbs for neurological diseases is their low accessibility to the CNS compartment. The normal brain-to-blood IgG concentration ratio of IV infused mAbs is approximately 0.1%. The passage through the BBB could be facilitated by the use of bispecific Abs where one specificity recognizes a receptor at the BBB, which promotes transcytosis, and the other specificity recognizes a potential therapeutic target such as Aβ, tau or tumor-specific targets (Figure 3). The best studied receptors for targeting brain tissue and promoting passage through the BBB are the insulin receptor (InsR), the LDL-related protein type 1 (LRP1) and the transferrin receptor (TfR) [178,179]. Using bispecific Abs with BBB shuttle function has been shown to increase brain-to-blood IgG concentration ratio of IV infused mAbs to 2–3% [180]. Other methods to improve mAb delivery to the CNS compartment are also being explored [181].

Interestingly, a recent double-blind trial investigated the effects of intrathecal and intravenous administration of rituximab versus placebo on a number of biomarkers of B cells depletion, inflammation and neurodegeneration in progressive MS (RIVITALISE trial; NCT01212094). The trial was discontinued early because at interim analysis, cerebrospinal fluid (CSF) B cells were only partially and transiently depleted and neurofilament light chain levels used as a marker of axonal damage were unchanged. The study identified low CSF levels of lytic complement factors and paucity of cytotoxic CD56dim NK cells as key contributors to decreased efficacy of intrathecally-administered rituximab [74].

## 6. Indications in Neurology

### 6.1. Multiple Sclerosis

MAbs have revolutionized treatment of both relapsing and progressive forms of multiple sclerosis (MS). Currently approved mAbs have shown their efficacy through phase III randomized controlled trials (RCTs) and are mainly used in the highly active forms of the disease, where their benefits clearly outweigh associated risks. Infliximab, a chimeric IgG1 mAb against tumor necrosis factor-alpha (TNF-α) was tested in a phase II trial but the trial had to be prematurely terminated due to increased relapse activity under infliximab treatment [182].

The first FDA approved mAb is natalizumab, a humanized antibody directed against α4β1 integrin (CD49d), a molecule expressed on the surface of lymphocytes and monocytes and interacting with brain endothelial VLA-4 in order to mediate their entry into the CNS parenchyma. Natalizumab has been a great success of the translational research as it proved to significantly reduce the relapse rate, disability progression and magnetic resonance imaging evidence of disease activity [57,178]. Natalizumab is currently being used as a second line agent in the treatment of highly active or rapidly evolving severe relapsing-remitting (RRMS) with excellent overall long-term risk-benefit balance [58].

With regard to cytokine targets, briakinumab, a human IgG1 mAb targeting Il-12 and 23 was examined in a phase II trial in RRMS. Although briakinumab significantly reduced the annualized relapse rate and number of gadolinium-enhancing lesions on brain MRI its efficacy was not deemed satisfactory for further development, compared to other agents [183]. Ustekinumab is another human IgG1 mAb targeting Il-12 and 23 tested in a phase II trial in RR-MS patients. Ustekinumab subcutaneous injections showed no effect on the cumulative number of gadolinium-enhancing lesions and the trial was terminated prematurely. The low concentrations of ustekinimab crossing the blood-brain barrier and its administration at a stage that may be considered past the decisive step of mobilization of a Th17 autoimmune reaction were considered as possible causes of its failure [184].

Alemtuzumab is a humanized monoclonal antibody selectively targeting CD52. Within minutes from infusion it depletes T and B cells through antibody-dependent cell-mediated cytolysis (ADCC) and complement-dependent cytotoxicity (CDC), which is followed by slow repopulation from hematopoietic precursor cells over several months with a distinct temporal pattern [161]. Alemtuzumab was the first monoclonal antibody that proved its efficacy against an active comparator (interferon-β1a) in a phase II trial [10] and two phase III trials [11,12] regarding clinical and MRI outcomes. It is indicated for relapsing forms of MS in patients who have had an inadequate response to two or more disease-modifying treatments (DMTs) according to the FDA [13] or for highly active relapsing-remitting MS despite treatment with at least one DMT or if the disease is worsening rapidly (EMA) [185].

Rituximab is a chimeric anti-CD 20 antigen mAb initially licensed for B-cell non-Hodgkin lymphomas resistant to other chemotherapy regimens [4]. CD20, is a 297 a.a. membrane-associated phosphoprotein present on all B cells which include pre-B cells, immature B cells, mature B cells, memory B cells, and a small fraction of T cells but not in stem cells, pro-B cells, and plasma cells [4]. Rituximab depletes circulating B cells but not B cells in the bone marrow or lymph nodes [4], promoting B cell lysis via antibody-dependent cellular cytotoxicity (ADCC), complement-dependent cytotoxicity (CDD), and phagocytosis by macrophages and neutrophils [73]. A phase II, double blind, trial involving 104 patients with RR-MS assigned to either rituximab or placebo showed that patients receiving rituximab had significantly fewer total and new gadolinium-enhancing lesions on MRI, and the proportion of patients in the rituximab group which exhibited at least one relapse was significantly reduced at week 24 (14.5% vs. 34.3% in the placebo group, *p* = 0.02) and week 48 (20.3% vs. 40.0%, *p* = 0.04) [75]. However, a randomized controlled phase III study has not been conducted with rituximab in MS patients to date. Furthermore, rituximab was the first CD20-depleting therapy to also be examined in a phase II/III trial in primary progressive MS (PPMS) patients [76]. Rituximab did not meet the defined primary endpoints, but this trial cleared the way for the exploration of ocrelizumab in this disease stage as it gave valuable clues regarding its efficacy in progressive disease [186]. Nevertheless, rituximab is extensively prescribed off-label, notably in Sweden where up to 53% of MS patients may be under rituximab [77].

Ocrelizumab is a humanized mAb approved by FDA in 2017 for the treatment of patients with relapsing or primary progressive forms of multiple sclerosis. It targets the CD20 antigen on B-cells and is the only intravenous anti-CD20 antibody that has been proven safe and efficacious in two randomized controlled phase III twin trials in which it was compared to subcutaneous interferon beta-1a at a dose of 44 μg three times weekly for 96 weeks. A statistically significant decrease in the annualized relapse rate by 46% in trial 1 and 47% in trial 2 was observed in the ocrelizumab-treated group compared to the interferon beta-1a group. The percentage of patients with confirmed disability progression at 12 and at 24 weeks was significantly lower with ocrelizumab and the mean number of gadolinium-enhancing lesions in T1-weighted magnetic resonance scans was 94% lower with ocrelizumab in trial 1 and 95% lower in trial 2, compared to treatment with interferon beta-1a [68]. Ocrelizumab is the first approved treatment for primary progressive MS as it has shown benefit in several efficacy measures including a significantly lower percentage of patients with confirmed disability progression at 12 and 24 weeks and a significantly lower percentage of brain volume loss in a phase III double blind, placebo-controlled trial [69].

Ofatumumab was approved by the FDA for MS in 2020. It is another anti-CD20 mAb, B-cell depleting DMT for MS. It has proven its efficacy and safety through two phase 3 double blind studies (ASCLEPIOS I and II) in which it was compared to teriflunomide [72]. Patients on ofatumumab exhibited a significantly lower annualized relapse rate in both trials and the percentage of patients with disability worsening confirmed at 3 and 6 months was also significantly lower with ofatumumab compared to teriflunomide [72]. Ofatumumab bears the important advantage of being the first self-administered, B cell targeting DMT in MS, delivered via an autoinjector pen, enabling patients to self-administer the treatment at home, avoiding visits to the infusion center, a particularly relevant advantage during the current COVID-19 pandemic [187].

Inebilizumab, is a humanized mAb targeting CD19 which is expressed on a wider lineage of B cells, including early pro-B cells and persisting through maturation to some short-lived plasmablasts and plasma cells spared by anti-CD20 agents [49,188,189]. Inebilizumab is at an early stage of development for relapsing MS, but in a phase I trial it has shown a trend towards a decrease in new/newly enlarging and gadolinium-enhancing lesions on brain MRI [49].

Daclizumab, a humanized antibody directed at IL2R-α (CD25) was originally approved for the prevention of renal allograft rejection. Daclizumab blocks the high affinity IL-2 receptors, which contain the α subunit (CD25). Medium-affinity receptors, on the other hand, consist of two β subunits (CD122) and are not affected by daclizumab. Its net effect is thought to be a suppression of T-cell responses and expansion of CD56bright natural killer cells [18]. It was tested in subcutaneous injections against placebo and interferon-β-1α and demonstrated efficacy in RRMS [19,20,21]. Nevertheless, the high affinity IL-2 receptor is also present on natural regulatory T cells (CD4CD25Foxp3 Tregs), which are decreased by 60% under daclizumab treatment [22]. This effect may explain the development of serious adverse reactions, including fulminant autoimmune hepatitis, which led to restrictions in its use only for patients who had not responded to two other disease-modifying therapies. Following reports of secondary autoimmune reactions, including cases of encephalitis it was voluntarily withdrawn from the market (EMA press release) [23].

Finally, opicinumab is a human monoclonal antibody that targets LINGO-1, a protein known to suppress remyelination and regrowth of transected axons. By blocking LINGO-1, opicinumab has been shown to promote remyelination in vivo [133]. In a phase II clinical trial, in patients with optic neuritis failed to reach significance in recovery of latency in visual evoked potentials, using the contralateral eye as a baseline, compared to placebo [134]. A phase II RCT of opicinumab as an add-on therapy to intramuscular IFN-β1a showed an inverted U-shaped dose response regarding the primary endpoint (percentage of participants with confirmed improvement over 72 weeks of treatment), but the treatment effect was not statistically significant [135]. However, some subpopulations of the study seemed to benefit from the treatment. Therefore, further research is needed to better assess the potential benefits of opicinumab [135].

### 6.2. Migraine

Four mAbs were recently approved by FDA as prophylactic treatments for migraine. All of them target the calcitonin gene related peptide (CGRP), a key mediator in the pathogenesis of the disease. Mabs are the only disease-specific and mechanism-based prophylaxis for episodic and chronic migraine. Erenumab is the only fully human mAb and targets the CGRP receptor whilst eptinezumab, fremanezumab and galcanezumab target the CGRP ligand [34,35,40,42,43,44,45]. The pooled percentage of patients that exhibited at least a 50% reduction in mean migraine-days per month in a meta-analysis of phase III trials of anti-CGRP mAbs in episodic migraine was 50.8% (95% CI 44.9%–56.6%) and 41.8% (95% CI 24.6%–60.1%) in phase III trials of chronic migraine [190]. Galcanezumab was also proven to be efficacious in cluster headache. [46]. Their favorable risk-benefit profile and high tolerability reflected in low drop-out rates observed in clinical trials paved the way for a new era in the preventive treatment of migraine [190,191]. Long-term open label studies exceeding 1 year for fremanezumab and galcanezumab and 5 years for erenumab indicated good tolerance and demonstrated sustained improvements in many efficacy measures, suggesting that the primary disadvantage of these mAbs is their high cost [36,41,47].

### 6.3. Neuromyelitis Optica Spectrum Disorder (NMOSD)

Neuromyelitis optica spectrum disorder (NMOSD), or Devic disease, is a chronic autoimmune condition in which a humoral response targets astrocytes leading to inflammatory demyelinated lesions affecting primarily the optic nerves, spinal cord and brainstem. In most cases NMOSD is associated with the presence of pathogenic anti-AQP4 antibodies [192]. The most commonly used disease-modifying treatments for NMOSD are azathioprine and rituximab [193]. Rituximab, an anti-CD20 mAb depleting B cells has shown efficacy in preventing relapses in several case series and retrospective analyses [78,79,80,81,82,83]. Inebilizumab, a humanized mAb targeting CD19, received FDA approval for the treatment of neuromyelitis optica spectrum disorder (NMOSD) in adult patients who are seropositive for immunoglobulin G autoantibodies against aquaporin-4 (AQP4-IgG) in June 2020 [50]. Tocilizumab is an anti-interleukin 6 receptor (IL-6R) antibody blocking IL-6R signaling [194]. Interleukin 6 (IL-6) production has been reported to increase in NMOSD and to enhance AQP4-IgG secretion. Studies have shown a favorable effect of tocilizumab in NMOSD patients who have failed to respond to other therapies [109,110,195]. Similar to tocilizumab, satralizumab is another humanized anti-IL-6 receptor IgG2 mAb licensed as a disease-modifying treatment for anti-AQP4 seropositive NMOSD on the basis of two successful phase III trials [107,108].

Another mAb proved to be efficacious in the treatment of NMOSD is eculizumab, a humanized antibody that reduced relapse rates from 43% to 3% in the treated group in patients with AQP4-IgG-seropositive NMOSD [28]. Autoantibodies against AQP4 are known to exert their cytotoxic action via complement activation [51,52]. Eculizumab inhibits the activation of terminal complement protein (C5) pathway by binding specifically and with high affinity to C5 [29]. Ravulizumab, a newer humanized mAb against C5 with less frequent infusion regimen compared to eculizumab is being evaluated for efficacy and safety in a Phase 3, placebo-controlled, open-label, multicenter study in adult patients with anti-AQP-4 (+) neuromyelitis optica spectrum disorder (NMOSD) [136]. A recent meta-analysis of clinical trials of eculizumab, inebilizumab, rituximab and satralizumab revealed that these mAbs significantly reduced annualized relapse rate (mean reduction −0.27, 95% CI: −0.36 to −0.18, *p* < 0.0001) and disability (mean Expanded disability status scale (EDSS) score reduction −0.51, 95% CI: −0.92 to −0.11, *p* = 0.01). In a subgroup analysis eculizumab was found more effective in decreasing on-trial relapse risk in anti-AQP-4+ patients [30].

Finally, aquaporumab is a nonpathogenic high-affinity recombinant human monoclonal antibody with slow washout competing with the pathogenic AQP4 autoantibody. Aquaporumab, which has not yet entered clinical trials, is a product of clonally expanded plasmablasts from the CSF of NMOSD patients with mutated Fc region to eliminate effector functions of complement-mediated cytotoxicity and antibody-dependent cell-mediated cytotoxicity [117,118].

### 6.4. Idiopathic Inflammatory Myopathies (IIM)

The idiopathic inflammatory myopathies (IIM) are a heterogeneous group of immune-mediated myopathies comprising of: dermatomyositis (DM), polymyositis (PM), inclusion body myositis, immune-mediated necrotizing myopathy and antisynthetase syndrome [196]. There is evidence of efficacy of mAbs in inflammatory myopathies. Rituximab was used in an open-label study of 6 patients with dermatomyositis refractory to previous treatments and resulted in clinical improvement in muscle strength, rash, alopecia, and forced vital capacity measurements, correlating with time of B cell depletion by rituximab. [84]. Similar results have been found in small open-label clinical trials involving polymyositis [85]. The Rituximab in Myositis (RIM) trial was a randomized double-blind, placebo-controlled trial of refractory juvenile and adult DM and PM patients. Although it did not meet its primary or secondary end points of efficacy, 83% of myositis patients met the clinical studies group definition of improvement [86]. In addition, rituximab had a steroid-sparing effect, it reduced the incidence of skin rashes and it was more beneficial in patients with myositis autoantibodies [87,88]. The report of increased tumor necrosis factor (TNF) levels in dermatomyositis and polymyositis has led to the trial of the TNF blocking agents etanercept and infliximab in both conditions [197]. A small pilot randomized, double-blind placebo-controlled trial in patients with refractory active DM and PM supported the efficacy of infliximab [198]. The results of tocilizumab, a mAb, which binds and inhibits both soluble and membrane -bound IL-6 receptors in a phase 3 trial in DM and PM are also expected shortly [111].

Inclusion body myositis (IBM) is an idiopathic inflammatory myopathy affecting the elderly. Bimagrumab-a fully human monoclonal antibody blocking the activin type II receptor (ActRII-A and ActRII-B) and preventing binding to their natural ligands (myostatin, activin and growth and development factor 11), was tried in individuals with inclusion body myositis in a randomized, double-blind, placebo-controlled phase 2b trial (RESILIENT) but failed to meet its primary end-point (increased 6-min walking distance) or improve muscle strength [199]. Alemtuzumab has been studied as a potential therapy in the treatment of inclusion body myositis in a small open label trial with promising results [200].

### 6.5. Myasthenia Gravis (MG)

Rituximab is commonly used in refractory cases of myasthenia gravis (MG) in which conventional immunomodulatory therapies have failed, even though evidence suggests that rituximab performs better in new-onset generalized MG than in cases that have become refractory to conventional immunosuppressants [89]. Uncontrolled studies have provided evidence of efficacy in several measures of efficacy such as clinical improvement, time to relapse, reduction in steroid use [90], decrease in antibody titers [91] and reduced in-hospital costs in a proportion of MG patients [92,93,94,95,96,97]. The efficacy of rituximab may be more pronounced in anti-Musk Ab MG, in which clinical improvement is associated with significant reduction in anti-Musk Ab titers, even to levels below detection [94,95,97,98]. Several empirical rituximab dosing regimes have been used; fixed repeat infusions every 3 or 6 months, repeat infusions when there is clinical exacerbation and others suggest using peripheral blood CD27+ memory B cells as a biomarker of impending MG reactivation [99]. Other anti-CD20 mAbs already licensed for MS such as ocrelizumab or ofatumumab could prove even more beneficial in MG given their 100% human composition. However, they have not been tested for MG and they would still not overcome the limitation of all CD20 mAbs, which is that they do not target plasmablasts and plasma cells that do not express CD20. Survival of long-lived plasmablasts may explain why several MG patients do not respond to rituximab. Monoclonal Abs targeting CD19 (e.g., inebilizumab) or CD38 (TAK-079) also expressed on some plasma cells could theoretically outperform rituximab but no data on the effects of this strategy exists yet [201].

On the other hand, eculizumab, which is a humanized mAb against C5 complement protein, originally used to treat paroxysmal nocturnal hemoglobinuria (PNH) is an approved treatment option for generalized AChR antibody-positive MG. Eculizumab inhibits the C5 convertase and thereby limits the formation of the terminal complement lytic complex [29,31]. It is safe and efficacious for refractory MG. REGAIN, a phase 3 double–blind, placebo-controlled study of eculizumab enrolled 125 treatment-refractory AChR+ patients with generalized MG of moderate to severe severity at 72 centers in Asia, Europe, Latin and North America. The primary endpoint, the mean ranked difference in the change in myasthenia gravis activities of daily living (MG-ADL) score between baseline and placebo at week 26 was not met despite significant change in 18 of 21 secondary efficacy measures. Improvement in MG-ADL was noted from the first week after infusion, it was maximal around 12 weeks, and was maintained for the duration of the 130-week observation [32]. Nevertheless, this is only relevant to AchR antibody + MG as in most MuSK+ cases damage is not mediated via complement pathway activation and its efficacy in double seronegative MG is unknown [91]. In addition, genetic variants of C5 have been shown to compromise response to eculizumab [33]. Life-threatening meningococcal infection is the most significant adverse effect of eculizumab, which necessitates vaccination against Neisseria meningitidis prior to treatment onset [32]. Ravulizumab, a newer humanized mAb against C5 is being tested in generalized MG [138], with the advantage of a less frequent infusion regimen (every 8 weeks instead of every 2 weeks in the case of eculizumab). Another promising target for MG is the CD40-CD40L interaction. Iscalimab, a fully human non cell-depleting mAb against CD40 blocks T cell-dependent antibody responses to both neo and recall antigens [202]. However, a double-blind, placebo-controlled, phase II trial in generalized MG did not show a statistically significant difference in QMG scores between the iscalimab group and placebo [203].

Blocking the neonatal FcRn receptor is yet another novel strategy for the treatment of MG. The FcRn binds to IgGs, including anti-AchR and anti-Musk antibodies thus preventing their degradation and leading to an increase in their titers. Rozanolixizumab, nipocalimab and batoclimab are all human mAbs that bind the FcRn, leading to a reduction in auto-antibody titers. In a phase 2a, randomized, double-blind, placebo-controlled trial, rozanolixizumab once-weekly SC infusions failed to demonstrate a significant change in QMG from baseline to day 29 despite a reduction in anti-AchR levels. Nevertheless, considering a range of pre-specified clinical efficacy measures (QMG, MG-ADL, and MGC), the data suggest rozanolixizumab has potential to provide clinical benefit in patients with moderate-to-severe generalized MG and was well tolerated [140]. Nipocalimab (M281) has also completed a phase II study of generalized MG with positive results and the batoclimab (HBM9161) phase II trial is currently recruiting [119,132,204]. Efgartigimod is an investigational antibody fragment targeting the neonatal Fc receptor (FcRn) with positive results in completed phase II and III trials of generalized MG [125,126]. In the phase II trial all patients treated with efgartigimod showed a rapid decrease in anti-AChR autoantibody levels and 9 out of the 12 efgartigimod-treated patients exhibited a rapid and long-lasting improvement in all 4 measures of efficacy (Myasthenia Gravis Activities of Daily Living, Quantitative Myasthenia Gravis, and Myasthenia Gravis Composite disease severity scores, and revised 15-item Myasthenia Gravis Quality of Life scale) [125].

### 6.6. Immune-Mediated Peripheral Neuropathies

Rituximab has been tried in a number of peripheral neuropathies, which are thought to be antibody-mediated and do not respond to the administration of intravenous immunoglobulins or require very frequent infusions. In multifocal motor neuropathy (MMN), a rare, symmetric, demyelinating, purely motor neuropathy, rituximab has had conflicting results: one case report showed yearly rituximab infusions resulting in reduction of IVIG dosage from every seven days to every 12 days over a five year period, [100] but another showed that in two patients with MMN, one had a decrease in total IVIG dosage while the other required an increase, whilst there was no significant clinical improvement, or change in Rankin disability scores [101].

In addition, anti-myelin associated glycoprotein (anti-MAG) neuropathy, a chronic sensorimotor demyelinating polyneuropathy is another entity in which rituximab has been tested. Open-label studies indicate that 30–50% of patients respond to rituximab [205] and two double-blind placebo-controlled trials confirmed these findings [102,103]. In a double blind, placebo controlled RCT of rituximab in anti-MAG neuropathy, four of 13 patients treated with rituximab showed improvement in leg disability scores whereas none of 13 placebo patients showed improvement. Also, there was a significant reduction in time to ten-meter walk in the rituximab group [102]. Gazzola et al. retrospectively also found that rituximab was effective in 10/33 patients and that the beneficial response lasted 42 ± 23 months after an average 5-year follow-up [104].

With all available treatments for chronic inflammatory demyelinating polyneuropathy (CIDP), one in three cases remains refractory, indicating that there is a need for efficacious alternatives [206]. Rituximab has been tried in CIDP with some case reports suggesting a favorable response [85,101]. Muley et al. in a small retrospective study of 11 patients with refractory CIDP described a rapid and in many cases impressive response, indicating that rituximab may be a useful alternative to established treatments [105]. A subcutaneous efgartigimod phase II study in adults with CIDP (ADHERE trial) has recently commenced recruiting [127]. Interestingly, eculizumab was examined in a phase II, randomized, placebo-controlled, masked trial of in 34 subjects with Guillain–Barré syndrome, which indicated that eculizumab was safe but it did not achieve a clinical measure of efficacy [207].

### 6.7. Neurooncology

Advances in our understanding of the genetic and cellular changes that drive carcinogenesis in the brain have been translated into new treatment targets. Targeting cell-signaling pathways with mAbs is a promising strategy in oncology. However, in the case of brain tumors the BBB is a special concern as it may prevent therapeutic antibody entry to the parenchyma [208]. Bevacizumab, a humanized recombinant monoclonal antibody that targets vascular endothelial growth factor (VEGF), has been shown to be well tolerated and efficacious in delaying tumor progression in the treatment of recurrent malignant glioma and is FDA approved for recurrent glioblastoma [14,15,16,209]. Rilotumumab, a fully human IgG2 anti-hepatocyte growth factor (HGF) mAb, preventing activation of the c-Met receptor and tumor cell growth was not associated with significant antitumor activity in patients with recurrent glioblastoma in a phase II study [138]. A more recent phase II trial of rilotumumab combined with bevacizumab failed to significantly improve objective response compared with bevacizumab alone [17].

Preclinical safety data of a fully human, CD3-binding bispecific antibody (hEGFRvIII-CD3-bi-scFv) for immunotherapy of malignant glioma have been reported [210]. The hEGFRvIII:CD3 bi-scFv mAb comprises of two single chain antibody fragments (bi-scFvs) that bind mutant epidermal growth factor receptor variant III (EGFRvIII), a mutation frequently seen in malignant glioma, and human CD3ε on T cells and aims to promote T cell mediated destruction of glioma cells [211].

### 6.8. Alzheimer’s Disease (AD)

Over the past three decades, our efforts to discover neuroprotective disease-modifying treatments for Alzheimer’s disease (AD) have been dominated by the amyloid hypothesis [212]. Among other treatments aiming to reduce the load of Aβ in the brain parenchyma mAbs have been employed to target and promote Aβ clearance. Ponezumab, a humanized mAb against Aβ failed to show clinical benefits in a phase II trial and its development was discontinued [213]. Three anti-Aβ mAbs failed to show benefit in phase III trials and were terminated early; bapineuzumab and solanezumab in mild to moderate AD [214,215] and crenezumab in prodromal and mild AD [216]. Interestingly, in bapineuzumab trials, a spectrum of imaging alterations were observed on MRI, termed: amyloid-related imaging abnormalities (ARIA). These include FLAIR signal abnormalities thought to represent parenchymal vasogenic edema and sulcal effusions (ARIA-E), and signal changes on GRE/T2* sequences thought to represent microhemorrhages and hemosiderosis (ARIA-H). ARIAs are commonly asymptomatic and evidence suggests that they are associated with transient increases in vascular permeability and amyloid clearance. The greater incidence of ARIAs with bapineuzumab, compared to solanezumab or crenezumab is probably because it binds to both soluble and insoluble forms of Aβ [217]. Gantenerumab is currently being tested in two phase III trials of prodromal and mild AD at doses higher than those used in a previous phase III trial which was terminated early for futility [128,129].

Aducanumab, a human mAb targeting aggregated forms of Aβ had some initial promising results showing a significant decrease of Aβ and potential slowing of cognitive decline in phase I trials [112], but two phase III trials in prodromal to mild AD discontinued early for futility in March 2019 [113]. However, subgroup analysis of data from patients treated with high dose aducanumab in one of the two phase III trials (EMERGE trial) showed a 23% reduction in cognitive decline on the Clinical Dementia Rating Scale–Sum of Boxes (CDR-SB) score, along with a 27% reduction on the AD Assessment Scale–Cognitive Subscale 13 Items (ADAS-Cog-13) and a 40% reduction on the AD Cooperative Study–Activities of Daily Living Inventory for Mild Cognitive Impairment (ADCS-ADL-MCI) [114,115], keeping aducanumab, which is under consideration for FDA approval on track [116].

Donanemab (LY3002813), a humanized anti-Aβ is currently in phase 2 clinical trial to evaluate the safety, tolerability, and efficacy of donanemab in mild AD. Although at its original design this trial included an arm treated with donanemab in combination with a BACE 1 inhibitor (LY3202626), to inhibit the production of beta-amyloid, this treatment arm was dropped due to poor results of the BACE inhibitor in other trials. Completion of this study is expected in November of 2021. BAN2401 showed to be safe and probably efficacious in Aβ load and slowing cognitive deterioration in a phase I and II trial [122,123]. Since March 2019, BAN2401 is in recruitment of a phase III trial enrolling prodromal to mild AD patients [124]. 

The lack of clear proof of efficacy of Aβ targeting therapies so far has raised skepticism regarding the validity of the amyloid hypothesis, driving researchers to explore tau pathology as a plausible therapeutic target, particularly as cognitive decline in AD exhibits a better correlation with tau accumulation than with Aβ deposition [218,219,220,221]. Monoclonal antibodies targeting abnormal forms of tau protein and particularly soluble oligomers which appear to be the most neurotoxic form of *p*-tau [222] are being explored for efficacy in AD. To date, gosuranemab, zagotenemab, tilavonemab, and semorinemab are in Phase II trials of prodromal to mild AD [130]. RG7345, UCB0107, JNJ-63733657 and BIIB076 are other anti-tau mAbs at phase I clinical trials with RG7345 having already been discontinued [223]. Gosuranemab has also been tested in a phase II trial in progressive supranuclear palsy (PSP), another tauopathy manifesting with vertical gaze palsy, gait instability, other extrapyramidal signs and dementia, but failed to meet its primary efficacy end-point, leading to its discontinuation (PASSPORT trial) [131].

### 6.9. Parkinson’s Disease (PD)

There are no approved therapies that can modify the progressive course of Parkinson’s disease (PD). α-Synuclein is a core component of Lewy bodies and neurites, a sine qua non pathological feature of PD. a-Synuclein mutations are causative for some cases of familial PD and other lines of evidence support a key role of α-synuclein in PD pathogenesis [224]. Accumulation and aggregation of α-synuclein protein is observed throughout the nervous system in PD. Recent experimental data suggest that PD progression may arise due to spreading of pathological forms of extracellular α-synuclein throughout the brain via a cellular release, uptake and seeding mechanism. Cinpanemab, a recombinant humanized anti-α synuclein IgG1 mAb targeting aggregated α-synuclein is currently in Phase 2 trial (BIIB054) [120], which followed a single ascending dose phase 1 study [121].

Another high affinity α-synuclein mAb, (MEDI1341), which binds both monomeric and aggregated forms has been shown to sequester extracellular α-synuclein and attenuate its spreading in vivo. After intravenous injection into rats and cynomolgus monkeys, MEDI1341 rapidly enters the central nervous system and lowers free extracellular α-synuclein levels in the interstitial fluid (ISF) and CSF compartments. In a lentiviral-based in vivo mouse model of α-synuclein spreading in the brain, treatment with MEDI1341 significantly reduced α-synuclein accumulation [225]. MEDI1341 is now in Phase 1 clinical trial with the aim to develop it as progression modifying treatment for PD and probably also other synucleopathies.

### 6.10. Duchene’s Muscular Dystrophy (DMD)

Monoclonal antibody-mediated blockade of myostatin, a member of the transforming growth factor-β (TGF-β) family of ligands, has been shown to increase muscle mass and volume in wild type mice and non-human primates and to increase muscle mass and improve function in murine models of DMD [226]. However, a phase 2 randomized placebo-controlled trial of domagrozumab, a humanized anti-myostatin mAb in 6 to 16 year-old children with DMD did not exhibit a significant treatment effect in its primary efficacy measure (time to 4 stair-climb) [227].

## 7. Safety Considerations of mAbs

Although mAbs have changed the treatment landscape in many neurological diseases, their ever-increasing use has been associated with several immune-mediated and other adverse reactions [228]. The development of fully human mAbs has significantly reduced their immunogenic potential and it has improved their tolerability, compared to earlier chimeric or humanized mAbs [229]. Nevertheless, even human mAbs maintain the potential for adverse reactions, such as anaphylactic reactions and infusion related reactions (IRRs) [230]. Given the considerable overlap in manifestations of immunologically-mediated reactions it is frequently difficult to distinguish them on clinical grounds [231].

### 7.1. Infusion-Related Reactions (IRRs)

Infusion reactions are among the most common adverse events of mAb administration. IRRs are defined as “any signs or symptoms experienced by patients during the infusion of pharmacologic or biologic agents or any event occurring on the first day of drug administration [231]. Manifestations are typically related in time to drug administration and may range from pyrexia, pruritus, rash, to dyspnea, generalized edema and cardiac arrest [230]. Mild IRRs are considered common and most infusion protocols include strategies to prevent or minimize the severity of IRRs by prophylactic administration of antipyretics, antihistaminics and corticosteroids. IRRs manifest within 24 h, but they occur most frequently from 10 min to 4 h from onset of administration [229]. When these reactions appear and depending on their intensity and severity, infusion may have to be slowed down or stopped and the manifestations may have to be specifically managed.

### 7.2. Anaphylactic Reactions

True anaphylactic reactions require the development of anti-mAb antibodies of the IgE isotype. According to the Joint Task Force on Practice Parameters anaphylaxis is defined as “an immediate systemic reaction that occurs when a previously sensitized individual is re-exposed to an allergen (2010) [232]. Given that an initial exposure to an antigen is required for IgE production, anaphylactic reactions are not expected during the first mAbs infusion except in the rare case of pre-existing IgEs cross-reacting with the infused mAb [233]. Anti-mAb IgEs typically mediate dyspnea, chest tightness, hypotension, bronchospasm, and urticaria. Even fully human mAbs can cause allergic reactions due to the presence of carbohydrate moieties on their heavy chain [233]. Anaphylactoid reactions or non-allergenic anaphylaxis are defined as those reactions resembling the clinical picture of anaphylaxis but are not IgE mediated. They rather occur through a direct nonimmune-mediated release of mediators from mast cells and/or basophils or result from direct complement activation [234,235]. Complement activation-related pseudoallergy (CARPA) is a form of anaphylactoid reaction, resulting from activation of the complement system and release of C3a, C5a and C5b-9 anaphylatoxins, which trigger degranulation of mast cells and basophils. Rituximab and infliximab are among mAbs that may cause CARPA [236,237].

### 7.3. Cytokine Release Syndrome (CRS)

Cytokine release syndrome (CRS) is a systemic inflammatory response associated with certain infections and medications. Unlike immune-mediated hypersensitivity reactions, the development of the cytokine release syndrome (CRS), is largely dependent on the cell load and cell type targeted by the mAb rather than its allergenic properties [238]. MAbs activating T cells are most likely to cause CRS, which occurs when large amounts of pro-inflammatory cytokines are released by activated white blood cells, including B cells, T cells, natural killer cells, macrophages, dendritic cells, and monocytes [239]. It may have a widely varied presentation ranging from mild, flu-like symptoms to severe life-threatening overshooting inflammatory response with circulatory shock, vascular leakage, disseminated intravascular coagulation, capillary-leak syndrome, hemophagocytic lymphohistiocytosis-like syndrome and multi-organ system failure [239]. Severe CRS may be associated with cytopenias, elevated creatinine and liver enzymes, deranged coagulation, and inflammatory parameters such as elevated sedimentation rate of erythrocytes (SRE) and C-reactive protein (CRP) [240]. In many respects CRS can be considered an extreme form of an infusion reaction even though CRS may be delayed by days or even weeks after infusion. Severe life-threatening CRS have been described for mAbs used to treat hematological malignancies such as rituximab and alemtuzumab, which are also indicated as DMTs for multiple sclerosis [241]. Prophylactic infusion protocols as in the case of rituximab, ocrelizumab and alemtuzumab include corticosteroids aiming to prevent or minimize CRS.

### 7.4. MAb Immunogenicity and Neutralization

Mabs are sometimes recognized as allogenic and anti-drug antibodies (ADA) are formed against them. ADA formation may lead to mAb neutralization, rapid elimination and loss of efficacy, allergic reactions and increased cost of treatment. The more immunogenic the mAbs, the more likely the formation of ADAs, which explains why ADAs are more likely to form against chimeric than human mAbs, including infliximab and adalimumab [242]. Despite the greater similarity of humanized mAbs to homologous mAbs these proteins keep a potential immunogenicity especially when used as monotherapy. In the case of the anti-CD49d mAb natalizumab, ADAs have been identified in up 9% of MS patients of whom in 6% the presence of ADAs was permanent [58]. Patients with ADAs often experience breakthrough relapses, free natalizumab is no longer detectable and its target antigen (CD49d) becomes upregulated [59,60]. Evidence suggests that high titers of ADAs against natalizumab are highly indicative of permanent anti-natalizumab immunization whereas low levels are transient [37,38,61,62]. On the other hand, in the case of alemtuzumab, 29% of patients in CARE-MS I/II had developed anti-alemtuzumab serum antibodies after 1 year, with no evidence of loss of efficacy [11,12]. Similarly, in the clinical trials of erenumab, a human anti-CGRP receptor mAb, 2–8% of patients had developed ADAs but only a small percentage of patients were reported to have neutralizing anti-erenumab antibodies and their presence was not associated with reduced efficacy or increased incidence of adverse events [35,37,38,39]. Likewise, in clinical trials of the anti-CGRP peptide mAb galcanezumab ADAs were detected in 2.6–12.4% of patients and their titer did not impact galcanezumab concentrations, calcitonin gene-related peptide concentrations, or galcanezumab efficacy [48]. Neurologists should be aware of the possibility of development of ADAs, which in some cases may explain treatment failure or breakthrough disease.

### 7.5. Opportunistic Infections

MAbs affecting immune function by depleting cell populations (e.g., alemtuzumab, rituximab, ocrelizumab) or by blocking immune cell migration through endothelial barriers (e.g., natalizumab) have been associated with the occurrence of opportunistic infections. Development of progressive multifocal leukoencephalopathy (PML) due to JCV infection in 3 MS patients in a phase III trial of natalizumab led to its withdrawal from the market, to be relaunched in June 2006 with the caveat that it would be used as monotherapy in patients with relapsing forms of MS [56,243]. The overall risk of developing PML seems to increase with the presence of anti-JCV antibodies, the duration of therapy (especially over 2 years) and the prior use of immunosuppressants and ranges from 0.07 per 1000 cases in JCV (-) patients to 10 per 1000 in JCV (+) patients exposed to natalizumab for more than 61 months [244]. Extended interval dosing of natalizumab to approximately every 6 weeks instead of the approved every 4 weeks may be a de-risking strategy shown to lower the risk of PML, with evidence of maintaining clinical effectiveness [63]. PML has also been reported with other mAbs, including rituximab and ocrelizumab [70,245]. Natalizumab treatment has also been associated with cryptococcal meningitis and reactivation of latent tuberculosis [64,65]. Cases of reactivation of latent tuberculosis have also been reported with alemtuzumab treatment in MS patients and tuberculosis screening is therefore recommended pre-treatment [48]. In addition, Pasteurela infections, spirochete infections, esophageal candidiasis, cerebral nocardiosis, Listeria meningitis, Pneumocystis pneumonia and varicella-zoster virus (VZV) reactivation have also been reported with alemtuzumab in MS patients [246,247,248,249,250]. Both alemtuzumab and ocrelizumab were linked to a statistically significant increase in overall risk of infection, of mostly mild or moderate severity whereas infections were not increased to a statistically significant degree in natalizumab clinical trials [56,66].

### 7.6. Malignancies

A key role of the adaptive immune response is to tackle cancer development. Nevertheless, the effect of mAbs with immunocompromising or immunosuppressing action on the likelihood of developing malignancies is less than clear. In its phase III trial in primary progressive MS ocrelizumab, an anti-CD20, B cell depleting mAb reported 11 cases of malignancy in the active treatment arm of which four were breast adenocarcinomas [69]. Although the numbers do not support a statistically increased incidence of breast cancer, the summary of product characteristics (SPC) acknowledges that this possibility cannot be neglected and it is advised that women on ocrelizumab follow standard breast cancer screening per local guidelines [71]. Interestingly, in an observational open label study of rituximab, another anti-CD20 mAbs in patients with rheumatoid arthritis followed for 9.5 years there was no increased incidence of cancer [106]. Despite the uncertainties regarding the potential risk of carcinogenicity associated with immunocompromising mAbs used in neurology, which warrants further evaluation, the overall benefit-risk balance in the approved indication is probably not significantly impacted.

### 7.7. Secondary Autoimmunity

Mabs targeting immune-related epitopes have also been linked to the occurrence of various autoimmune disorders. Secondary autoimmune disease directed primarily against the central nervous system, liver, and skin resulted in the withdrawal of daclizumab in 2018. These were mainly in the form of eczematous skin lesions but also rash associated with eosinophilia and organ involvement (DRESS syndrome), fulminant hepatitis, autoimmune vasculitis and encephalitis with anti-NMDA and anti-GFAP auto-antibodies [24,25,26]. It is tempting to associate the targeting of the CD25 receptor present also on CD4+CD25+ FoxP3+ regulatory T cells and their consequent decrease with the occurrence of the above autoimmune conditions under daclizumab treatment [27,251].

Nevertheless, among mAbs used in neurology, secondary autoimmunity is most commonly encountered with alemtuzumab. Over a follow -up of up to 10 years, almost half of alemtuzumab-treated MS patients had developed some autoimmune condition [252,253]. The most frequently affected organ was the thyroid, with up to 29% of patients developing thyroiditis [254], followed by idiopathic thrombopenic purpura (ITP) [255], and Goodpasture Syndrome with autoantibodies against the glomerular basement membrane [256]. Many other autoimmune conditions have been reported with alemtuzumab including but not limited to immune-mediated neutropenia and autoimmune hemolytic anemia [257], diabetes mellitus type 1 [258], Still’s disease [259], myositis [260] and alopecia areata universalis [261]. Although most alemtuzumab-associated autoimmune conditions are autoantibody-mediated some others such as alemtuzumab-related vitiligo are T cell mediated [262]. How alemtuzumab triggers autoimmune diseases remains unclear. Following initial depletion, CD52+ T and B lymphocytes of different clonal specificities gradually reconstitute the adaptive immune system, with B lymphocytes exhibiting faster reconstitution and an overshooting response, which may explain the auto-antibody-mediated autoimmunity. Furthermore, there is evidence for a role of interleukin IL–21 in driving the proliferation of chronically activated, oligoclonal, effector memory T cells in autoimmunity following bevacizumab [263]. In addition, infliximab was found to exacerbate multiple sclerosis in a phase II trial leading its clinical development for MS to a halt [182] and CNS demyelinating disease is a recognized potential complication of the use of anti-TNF agents for the treatment of rheumatic and inflammatory bowel disease [264,265].

### 7.8. Summary of Safety

MAb-related adverse reactions may be predictable to some extent by their target specificity and mechanism of action but in many cases mAb-related adverse reactions remain unpredictable (e.g., natalizumab associated with hepatotoxicity) [67]. Occurrence of adverse events temporally and/or mechanistically associated with treatment administration, and their evolution following treatment discontinuation should raise suspicion of a possible adverse drug reaction. The clinical development program and post-marketing pharmacovigilance monitoring are the only guarantors of safety. Treating neurologists’ expertise in the use and implementation of risk-mitigation strategies of mAbs and vigilance are warranted.

## 8. Concluding Comments

The use of mAbs in neurological therapeutics is expanding rapidly. Many more mAbs are in different stages of development, suggesting that their use is likely to spread even more in the coming years. Advances in deciphering the molecular mechanisms of neurological disease drive the identification of novel plausible therapeutic targets. MAbs are characterized by exquisite target specificity along with numerous options of different mechanisms of action provided by contemporary molecular engineering technologies. These features make mAbs precision tools of unlimited potential to act on identified key pathogenetic targets.

Neurological indications of mAbs are no longer restricted to immunological targets. MAbs now have a primary role in the prophylactic treatment of migraine and are being developed as disease-modifying treatments for neurodegenerative conditions such as Alzheimer’s and Parkinson’s disease. It becomes imperative that neurologists acquire deep knowledge of their indications, potential side effects and strategies to minimize mAb-associated risks.

## Figures and Tables

**Table 1:** Marketed monoclonal antibodies used in neurology.

Name	Type	Target	Action	Route	Neurological Indication	Adverse Effects of Special Interest	References
Alemtuzumab	humanized IgG_1_	CD52	Depletes CD52^+^ T and B cells	IV	RR-MS *	Infusion reactions Secondary autoimmunity Cerebrovascular accidents	[7,10,11,12,13]
Bevacizumab	humanized IgG_1_	VEGF	Inhibition of angiogenesis	IV	Glioblastoma *	hypertension, gastrointestinal perforation, bleeding, PRES	[14,15,16,17]
Daclizumab	humanized IgG_1_	IL2R-α (CD25)	Blocks the high affinity IL-2 receptor containing the α subunit	SC	RR-MS *	Autoimmune encephalitis, hepatitis and rashes	[18,19,20,21,22,23,24,25,26,27]
Eculizumab	humanized IgG_2/4_	C5 complement protein	Inhibition of the terminal C5 complement pathway	IV	Anti-AChR Ab^+^ MG * AQP-4^+^ NMOSD *	Meningococcal infections	[28,29,30,31,32,33]
Eptinezumab	humanized IgG_1_	CGRP ligand	Selectively bind to isoforms a and b of CGRP	IV	EM* and CM *	Nasopharyngitis Hypersensitivity reactions	[34]
Erenumab	fully human IgG_2_	CGRP receptor	Competitively and reversibly binds the CGRP receptor	SC	EM * and CM *	Constipation Injection site reactions	[35,36,37,38,39]
Fremanezumab	humanized IgG_2_	CGRP ligand	Selectively bind to isoforms a and b of CGRP	SC	EM * and CM *	Injection site reactions	[40,41]
Galcanezumab	humanized IgG_4_	CGRP ligand	Binds CGRP and prevents its biological activity	SC	EM * and CM * Cluster headache	Injection site reactions	[42,43,44,45,46,47,48]
Inebilizumab	humanized IgG_1_	CD19	Depletes B cells and some short-lived plasmablasts and plasma cells	IV	AQP-4^+^ NMOSD	Infusion reactions, infections	[49,50]
Infliximab	chimeric IgG_1_	TNF-α blockade	TNF-α signaling blockade	IV	DM/PM Behcet disease Neurosarcoidosis	Infusion reactions CNS demyelination	[29,51,52,53,54]
Natalizumab	humanized IgG_4_	α4β1 integrin (CD49d)	Inhibits the entry of lymphocytes into the brain parenchyma	IV	RR-MS *	PML, hepatotoxicity	[55,56,57,58,59,60,61,62,63,64,65,66,67]
Ocrelizumab	humanized IgG_1_	CD20	Depletes B cells	IV	RR-MS * PP-MS *	Infusion reactions, infections	[68,69,70,71]
Ofatumumab	fully human IgG_1_	CD20	Depletes B cells	SC	RR-MS *	Injections site reactions, infections, neutropenia	[8,72]
Rituximab	chimeric IgG_1_	CD20	Depletes B cells	IV	RR-MS NMOSD; MG; CIDP; MMN, anti-MAG neuropathy PM/DM	Infusion reactions PML	[4,73,74,75,76,77,78,79,80,81,82,83,84,85,86,87,88,89,90,91,92,93,94,95,96,97,98,99,100,101,102,103,104,105,106]
Satralizumab	humanized IgG_2_	IL-6 receptor	IL-6 receptor signaling blockade	SC	Anti-AQP4 Ab^+^ NMOSD *	Infections, neutropenia, elevated liver enzymes	[107,108]
Tocilizumab	humanized IgG_1_	IL-6 receptor	IL-6 receptor signaling blockade	IV	NMOSD CRS	Infusion reactions, Infections	[9,109,110,111]

*: officially approved indication, AQP4: aquaporin 4; CIDP: chronic inflammatory demyelinating polyneuropathy; CGRP calcitonin gene-related peptide; CNS: central nervous system; CM: chronic migraine; CRS: cytokine release syndrome; DM/PM: dermatomyositis/polymyositis; EM episodic migraine; IL-6R: interleukin 6 receptor; MG: myasthenia gravis; MMN: multifocal motor neuropathy; NMOSD: neuromyelitis optica spectrum disorder; PML: progressive multifocal leukoencephalopathy; PP-MS: primary progressive multiple sclerosis; PRES: posterior reversible encephalopathy syndrome; RR-MS: relapsing remitting multiple sclerosis; TNF-α: tumor necrosis factor-α.

**Table 2:** Monoclonal antibodies in development for various neurological indications.

Name	Type	Target	Action	Stage of Development	Neurological Indication	References
Aducanumab (BIIB037)	fully human IgG1	Aβ	Binding of the aggregated Aβ forms	In phase IΙΙ	Prodromal to mild AD	[112,113,114,115,116]
Aquaporumab	fully human (mutated Fc)	AQP-4	Competitively inhibits binding of anti-AQP-4 auto-Abs	not yet in clinical trials	NMOSD	[117,118]
Batoclimab (HBM9161)	fully human IgG1	FcRn	Reduction of auto-antibody levels	In phase II	MG	[119]
Cinpanemab (BIIB054)	humanized IgG1	α-synuclein	Prevention of accumulation and aggregation of α-synuclein	In phase II	PD	[120,121]
Donanemab (N3pG)	humanized IgG1	Aβ	Binding aggregated Aβ forms	In phase II	Mild AD	[122,123,124]
Efgartigimod	Antibody fragment	FcRn	Reduction of auto-antibody levels	In phase II for CIDP completed phase III for MG	MG CIDP	[125,126,127]
Gantenerumab (RG1450)	fully human IgG1	Aβ	Binding aggregated Aβ forms	In two phase III trials	Prodromal and mild AD	[128,129]
Gosuranemab (BIIB092)	humanized IgG4	tau	Targeting abnormal forms of tau protein or soluble oligomers	In phase II	Prodromal to mild AD	[130,131]
Nipocalimab (M 281)	fully human IgG1	FcRn	Reduction of auto-antibody levels	Completed phase II trial	MG	[132]
Opicinumab (BIIB033)	fully human IgG1	LINGO-1	Promotion of remyelination	In phase II	MS	[133,134,135]
Ravulizumab (ALXN1210)	humanized IgG2/4	C5	Inhibition of the C5 terminal complement pathway	In phase III	AQP-4^+^ NMOSD, MG	[136,137]
Rilotumumab (AMG102)	fully human IgG2	HGF	Prevents activation of the c-Met receptor and tumor cell growth	In phase II	Glioblastoma	[17,138]
Rozanolixizumab (UCB 7665)	humanized IgG4	FcRn	Reduction of auto-antibody levels	Completed a phase II study	MG	[139]
Semorinemab (RG6100)	humanized IgG4	tau	Targeting all isoforms of tau protein	In phase II	Prodromal to mild AD	[130]
Tilavonemab (ABBV 8E12)	humanized IgG4	tau	Targeting abnormal extracellular forms of tau protein	In phase II	Prodromal to mild AD	[130]

Aβ: amyloid beta peptide; AD: Alzheimer’s disease; AQP4: aquaporin 4; CIDP: chronic, FcRn: neonatal Fc receptor; HGF: hepatocyte growth factor; LINGO-1: Leucine rich repeat, Ig domain containing, Nogo receptor interactive protein-1; MG: myasthenia gravis; NMOSD: neuromyelitis optica spectrum disorder.
